# Predicting the Adoption of a Sustainable Diet in Adults: A Cross-Sectional Study in Italy

**DOI:** 10.3390/nu15122784

**Published:** 2023-06-17

**Authors:** Beatrice Biasini, Alice Rosi, Francesca Scazzina, Davide Menozzi

**Affiliations:** Department of Food and Drug, University of Parma, Parco Area delle Scienze, 27/A, 43124 Parma, Italy; beatrice.biasini@unipr.it (B.B.); alice.rosi@unipr.it (A.R.); francesca.scazzina@unipr.it (F.S.)

**Keywords:** diet sustainability, Mediterranean diet, theory of planned behavior, dietary habits, adult population

## Abstract

Shifting food choices towards sustainability entails the analysis of dietary behavior determinants. This study aimed to explain and predict the intention to follow a sustainable diet and its adoption in a representative sample of adults (*n* = 838) in Italy. An online survey based on the theory of planned behavior (TPB) was developed. The adoption of a sustainable diet was measured as self-perceived behavior, adherence to the Mediterranean diet (MD), and food consumption frequencies. Psychometric analysis and correlations between attitude, subjective norms, and perceived behavioral control (PBC) with both intention and behavior assessments were evaluated. Structural equation models were used to test whether and to what extent attitude, subjective norms, and PBC explained intention and behavior. Significant associations were found between TPB constructs and the behavior measures underlining the main role of intention and PBC in affecting behavior. The applied TPB models explained behavioral intention at most (78%). The results suggested promising interventions in narrowing the attitude–behavior gap to encourage specific adult population groups in Italy to adopt virtuous food consumption habits. Beside the implementation of price mechanism strategies, educational initiatives to increase awareness about food and diet sustainability issues and the reinforcement of the perceived control on food consumption at the individual level are recommended.

## 1. Introduction

Consumer behaviors form diets that, in turn, not only shape nutrition and health outcomes based on their constituent attributes, such as quantity, quality, diversity, safety, and adequacy, but also mediate broader impacts on the economy, social equity, and the environment [1]. If sub-optimal diets, primarily low in whole grains and high in salt, emerge as the third leading risk factor for mortality worldwide [2,3], several plant-based diets can improve cardiovascular health [4,5] and reduce the non-communicable diseases risk [6]. On the other hand, food systems are responsible for environmental exploitation, mostly due to the farm stage of production [7]. They play a major role in public health, which is strictly linked to food security and food safety, and affect the income level and distribution of a population, making diet affordability a significant challenge [8]. 

The extensive existing literature has shown positive associations between the adoption of a Mediterranean dietary pattern and cardiovascular [9], metabolic [10], cognitive [11], and bone [12] health, involving different life stages, while a low adherence to the Mediterranean diet (MD) has been associated with an increased risk of cancer and mortality [13]. Similarly to the global reference diet proposed by the EAT Lancet Commission [14], the MD is characterized by a daily consumption of plant-based foods such as whole grains, fresh produce, and vegetable oils rich in unsaturated fatty acids. However, the inclusion of modest quantities of fish, meat, and dairy foods is optional in the diet described by the commission. The MD benefits human and environmental health [15] by reducing the use of natural resources and mitigating climate change [16,17,18]. Moreover, as part of the Intangible Cultural Heritage of Humanity [19], it refers to socio-cultural values and promotes profitability for local farmers. 

Several conditions, including food availability, physical and economic accessibility, policy framework, and culture, as well as personal attitude and food preference, variably affect food intake [1,20,21]. 

Exploring consumers’ intentions, and relative antecedents, to engage in sustainable dietary choices is pivotal for the implementation of successful strategies aimed at driving such transition by addressing the so called “attitude–behavior gap”, which reflects the discrepancy between having a favorable attitude toward a certain behavior and the behavior itself [22]. In this regard, theoretical models can be used to recognize relevant factors facilitating or preventing sustainable food consumption and on which build fruitful approaches enabling the adoption of sustainable dietary behavior. Among these, the theory of planned behavior (TPB) aims to understand human behavior, using intention and perceived behavioral control (PBC) as proximal determinants of a person’s behavior. Intention, indicating the conscious motivation to exert effort to engage in a behavior, is in turn predicted by the subject’s attitude towards the behavior (i.e., positive or negative evaluations of enacting the behavior), the subjective norm (i.e., perception about whether significant others think the subject should or should not perform the behavior), and PBC (i.e., the subject’s expectancy that putting in action the behavior is under his/her control). 

Each TPB construct results from prior determinants. Specifically, attitudes arise from personal beliefs related to the behavior’s implications; subjective norms depend on significant others’ approval or disapproval about the possibility that the subject will engage in that specific behavior; PBC is driven by the self-perception of being able to perform a given behavior successfully [23]. According to the TPB, interventions designed to change behavioral, normative, or control beliefs may successfully modify behavior in the desired direction by acting upon intention, attitudes, subjective norms, and PBC. Behavior is reasoned or planned as it is assumed that a person’s intention and behavior consistently arise from prior determinants, i.e., beliefs, even irrational ones. Other studies have shown the impact of deviations from rationality on consumers’ values for health and environmentally sustainable food products [24]. In this context, self-identity, previously defined as the “salient part of an actor’s self that relates to a specific behavior” [25], can have a relevant role in affecting intentions and actions by acting as an additional predictor [26,27] or as a moderator [28] of the relationships predicted by the model [23]. 

The TPB has been applied to both healthy [29,30,31] and health-harming eating behaviors (e.g., alcohol use) [32]. A large number of studies have focused on the TPB applied to healthy eating, both considering certain dietary patterns (e.g., following a low-fat diet) [33,34,35,36] and discrete eating behavior (e.g., increasing fruit and vegetable consumption) [37,38,39,40,41]. To the best of our knowledge, however, only a few studies referred to more than one sustainability perspective (e.g., health and the environment) by applying the TPB model in relation to a diet-related behavior [42,43]. In parallel, however, the adherence to the MD has been proposed as target behavior in previous studies applying the TPB [44,45]. Therefore, this study tried to embrace multiple diet sustainability dimensions, consistent with the sustainable diet(s) definition provided by the FAO [46], by using the TPB as a theoretical framework. 

The aim of this study was to explain and predict both the intention to adopt a sustainable diet (SuDiet) and the behavior of following a SuDiet in a representative sample of the Italian adult population. The study predicted the behavior assessed according to different outcome measures: (i) the self-perceived behavior; (ii) the overall MD adherence score, investigated as a proxy of actual sustainable dietary behavior; and (iii) the consumption frequency of single food groups. It was hypothesized that attitude, subjective norm, and PBC have a positive influence on the intention to eat sustainably, which in turn, with PBC, would predict behavior. Moreover, the moderating role of self-identity on all relationships between TPB antecedents, intention, and behavior was tested. 

## 2. Materials and Methods

### 2.1. Sample

In July 2019, a marketing company (i.e., Qualtrics International Inc., Seattle, WA, USA and Provo, UT, USA) distributed the online survey to a representative sample of adults residing in Italy (18–65 years), after receiving the approval from the local Institutional Ethical Committee (Comitato Etico Area Vasta Emilia Nord, 1139/2018/OSS/UNIPR). Informed consent was obtained from the respondents for being part of the study. Completing the survey took about 15 min. The parameters for subjects’ representativeness and the inclusion criteria for subjects’ enrolment have been described elsewhere [47]. Briefly, recruited subjects were representative of the adult population residing in Italy based on gender distribution, age range, and geographical areas of residence.

### 2.2. Measures

The subjects self-reported their socio-demographic variables. A SuDiet was defined according to the FAO’s statement [46] and explicitly defined as “a limited consumption of animal-based products, preference towards local and/or seasonal products that are respectful of environment and biodiversity” to provide the respondents with a unique interpretation. Before defining the SuDiet, the online survey included a question designed to understand the concepts the respondents associated more with the meaning of a sustainable diet (e.g., healthy and balanced diet; diet with a limited meat consumption). 

As suggested by Fishbein and Ajzen [23], we conducted qualitative interviews and focus groups for eliciting salient beliefs, such as expected outcomes, social norms, and the factors potentially affecting the adoption of the SuDiet. More specifically, a standard TPB-based questionnaire was developed based on the outputs obtained from: (i) a qualitative explorative questionnaire administered to postgraduate students (*n* = 22) with different academic backgrounds; (ii) one focus group performed with undergraduates in food science and technology (*n* = 6); (iii) interviews to adults (*n* = 10) of both genders and varying socio-demographic characteristics. 

Among the TPB constructs, attitude (i.e., positive or negative evaluations of adopting a sustainable diet in the near future), subjective norm (i.e., perception about whether significant others think the subject should or should not adopt a sustainable diet in the near future), and PBC (i.e., the subject’s expectancy that adopting a sustainable diet in the near future is under his/her control) were modelled as exogenous variables affecting the intention to adopt a sustainable diet in the near future; in turn, PBC and intention were the antecedent variables predicting self-perceived behavior (i.e., adoption of a sustainable diet in the near future). To inform the TPB constructs, direct and indirect measures were applied using specific items: three to four single items were used as direct measures of TPB constructs, whereas three to eight item pairs of scales, multiplied to obtain a unique score, were applied as indirect measures (i.e., the beliefs) [23]. Each item was assessed on a specific 7-point scale. A description of the TPB constructs measurement is provided in the supplementary material (Supplemental Table S1). 

In parallel, an individual’s self-identity was measured with a single item using a 7-point scale (absolutely not/absolutely yes): “I think to myself as a person interested to diet sustainability”, adapted from Fishbein & Ajzen [23]. To investigate the moderating role of self-identity on all relationships between TPB antecedents, intention, and behavior, three groups were defined based on the tertiles of the self-identity (SI) score: the Low-SI group (score ≤ 4); the Medium-SI group (score = 5); the High-SI group (score ≥ 6) (see also [38,48]).

Two measures were included to identify potential discrepancies between the self-perception of behavior and actual (self-reported) performance. The first indicator was measured by a score composed using three items on self-perceived behavior, e.g., “I can say I have limited the consumption of animal-based products within the last 3 months” (totally disagree/totally agree). The selection of the three items was based the definition of sustainable diets provided by the FAO [46]. Then, we provided a more objective measure of behavior using a validated food frequency questionnaire [49] which score the subject’s adherence to the Mediterranean diet (0 = minimal adherence; 9 = maximal adherence). The adherence to the MD, considered as an example of a SuDiet, was used to assess the sustainability of the actual dietary behavior of participants. Finally, we used as dependent variables the single scores of the consumption frequency of different food groups (e.g., milk and yoghurt, wholegrain bread and substitutes, red meat, meat products, etc.) to measure the effects of the TPB variables on these dietary habits.

### 2.3. Data Analysis

The internal consistency, validity, and reliability of the TPB constructs were tested using Cronbach’s alpha, factor loadings (λ), and composite reliability (CR), respectively. We applied confirmatory factor analysis (CFA) and computed the average variance extracted (AVE) to confirm the convergent validity of the constructs. The discriminant validity of the constructs was tested by comparing the square root of the AVE of each construct with the inter-construct correlation [50]. 

We computed Spearman’s rank-order correlations (ρ) to analyze the relationship between salient beliefs and (i) their relative direct measures (i.e., attitude, subjective norms, and PBC), (ii) intention, and (iii) behavior. 

A structural equation modelling (SEM) analysis was performed to test the TPB models, whereas multi-group SEM was used to explore the role of self-identity as a moderator on all relationships between TPB antecedents, intention, and behavior. SEM allowed us to test the effects of latent exogenous variables on latent endogenous variables. SEM estimated the paths from the latent to the observed variables (labelled as λ, estimated by the measurement model, CFA), the paths from the exogenous to the endogenous variables (e.g., attitude → intention, labelled as γ), and the paths from the endogenous variables to other endogenous variables (e.g., intention → behavior, labelled as β). For simplicity, these latter paths were labelled as B for the unstandardized coefficients, and β for the standardized coefficients. The goodness-of-fit of the models was tested by χ^2^ and degrees of freedom (df), Tucker-Lewis Index (TLI), comparative fix index (CFI), root mean square error of approximation (RMSEA), and standardized root mean square residual (SRMR), while the coefficient of determination (R^2^) was used to assess the explained variance of the endogenous variables. In particular, model adequacy and goodness is generally confirmed when CFI and TLI > 0.95, and SRMR and RMSEA < 0.08 [51]. An analysis of the residuals was performed to diagnose possible sources of misspecification. The measurement invariance across groups through configural and metric invariance (equal factor loadings) was based on changes in the model fit, i.e., Δχ^2^ and ΔCFI. The models were estimated using IBM^®^ SPSS^®^ Amos™ 24.0 software with the Bayesian routine [52].

## 3. Results

### 3.1. Participants’ Characteristics and Their Perceptions of a Sustainable Diet

The time taken to complete the survey was applied as a quality control. After eliminating low-quality records (i.e., completed in <5 min), responses from a total of 838 subjects were finally considered. The socio-demographic characteristics, health conditions, and food-related habits of the final sample have been previously described [47]. Briefly, gender was equally distributed (52% females), and almost half of the subjects (47%) were at least 45 years old. Most of the sample had reached the secondary education level and had a normal weight status; however, males who were overweight or obese were more prevalent than females with the same conditions. Females were more responsible than males in purchasing food and preparing meals [47]. In Supplementary Table S2, participants’ characteristics are presented in parallel with those of the Italian population.

When asked to indicate a maximum of three concepts associated with a SuDiet, respondents primarily recognized its environmental dimension and then its healthiness (Table 1).

### 3.2. Descriptive Analysis of the TPB Constructs and the Underlying Beliefs

The measures utilized in the present study were fully reliable and all scales had satisfactory internal reliability [53] (Table 2). Indeed, the measurement model showed proper item reliability and convergent validity, with factor loadings (λ), CR values, AVE values, and Cronbach’s α all overcoming the relative suggested thresholds. In addition, the TPB constructs have discriminant validity, as shown by comparing the square root of AVE with the correlations between constructs (Table 3).

Overall, the sample reported a positive attitude and intention. Upper intermediate values were selected for the perceived control over behavior and self-identity, while lower intermediate scores were reported for the adoption of a sustainable diet in the near future. In addition, the opinion of peers does not seem to affect the choice to adopt the behavior of interest, as demonstrated by the neutral scores associated with subjective norms. Adherence to the MD was medium (median: 4.0, IR: 3.0–5.0), as already described in Biasini et al. [47]. 

As displayed in Table S3, the most important outcomes of adopting a SuDiet are the positive impact on health and the environment; these received the highest composite median scores. Food habit modification and the improvement of culinary skills received the lowest median scores, although still positive. Moreover, subjects reported that the adoption of a SuDiet is quite unlikely to trigger the improvement of culinary skills. Among significant others, doctors, nutritionists, and experts emerged as the figures whose opinions were the most considered, as these statements obtained higher scores compared to those referring to family, friends, or institutions. Overall, the results indicate a weak impact of social pressure. The main factors more associated with behavior were the informative labels on products, price reduction, and a wider variety of food in collective catering.

### 3.3. Correlation Analysis between TPB Constructs

As shown in Table 3, all the TPB constructs showed significantly correlations between one another (*p* < 0.001). The highest correlation coefficient (ρ > 0.7) can be observed between intention and PBC. This means that respondents who were more willing to adopt a SuDiet were those who perceived themselves to have more control over their behavior performance. Intermediate correlation levels (0.4 < ρ > 0.7) between the TPB variables and behavior as self-perceived by the subjects show that those perceiving themselves to already have adopted a SuDiet have a more favorable attitude towards and stronger perceived ability and intention to perform the behavior in the future. On the other hand, weaker correlations (ρ < 0.4) can be observed between the TPB constructs and the behavior, as measured by the adherence to MD. 

Medium-to-low correlations (*p* < 0.001), ranging from ρ = 0.66 to ρ = 0.16, were found between salient beliefs and their relative direct measure (attitude, subjective norm, and PBC), intention, self-perceived behavior, and actual behavior, measured as adherence to MD (Table 4). Among these, the normative (family behavior) and behavioral control (free time available) dimensions reached the highest and the lowest correlations with their relative constructs, subjective norms, and PBC, respectively. Intention to follow a SuDiet was moderately correlated with family and partner behavior, sensorial satisfaction, food variety in collecting catering, and accessibility to more exhaustive labels. The latter, together with family behavior and the possibility to support the local economy and small- or medium-sized farmers, was also moderately correlated with self-perceived behavior.

### 3.4. Predicting Ability of the TPB Models 

Table 5 reports two TPB models predicting self-perceived behavior (Model 1), and actual behavior, measured as adherence to the MD (Model 2). The data fit well with the models, as shown by the global fit indices [51]. Both Model 1 and 2 explain 78% of the variance in intention to adopt a SuDiet, while the explained behavior variance accounted for 54% (Model 1) and 13% (Model 2), respectively. In both models, intention is significantly affected by all of the TPB constructs (*p* < 0.001), although PBC shows the strongest effect. In turn, behavior is significantly predicted by intention in Model 1 (*p* < 0.001), and by intention and PBC in Model 2 (with *p* < 0.01 and *p* < 0.05, respectively). In both cases, the magnitude of the unstandardized coefficients (B) indicates that the adoption of behavior (as self-perceived or measured in terms of adherence to the MD) is most strongly affected by intentions. 

The path coefficients between the frequencies of consumption of single food group intake and the relative TPB drivers (i.e., intention and PBC) were estimated by 15 different models (Supplemental Table S4). Fruit, vegetable, and legume consumption frequency showed the highest explained variance, at a value of 8%. A higher intention to adopt a SuDiet in the near future resulted in a lower consumption frequency of meat products and sugar-sweetened beverages and a higher consumption frequency of fruits and vegetables. A higher fish, pulse, and olive oil consumption frequency is driven by stronger perceived behavioral control.

### 3.5. The Role of Self-Identity as a Moderator

The three self-identity groups showed statistically different medians for all variables (Table 6).

**Figure 1:**
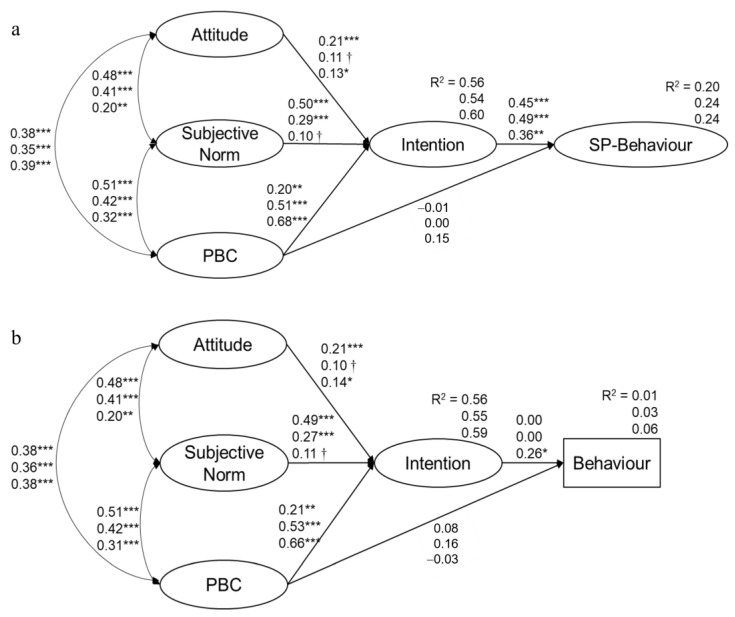
Multi-group SEM analysis with standardized regression coefficients and R^2^ (explained variance) for endogenous variables in Model 3 (**a**) and Model 4 (**b**). Upper values indicate standardized regression weights for low-self-identity group, middle values for medium-self-identity group, and bottom values for high-self-identity group. Such coefficients refer to the paths from exogenous to the endogenous variables (i.e., from attitude, SN, and PBC to intention and from PBC to (SP)-behavior), as well as to the path between endogenous variables (i.e., from intention to (SP)-behavior). *** *p* < 0.001, ** *p* < 0.01, * *p* < 0.05, † *p* < 0.10. PBC: perceived behavioral control; SP: self-perceived.

Initial testing of the hypothesized configural models with self-identity as a moderator yielded well-fitting results (Model 3 and 4, Table 7), showing that the predictors of intention changed when the groups were individually considered (Figure 1). The analysis also provided reasonable evidence in support of measurement invariance permitting a meaningful comparison between the groups in Model 3 (factor loading invariance: Δχ^2^ (26) = 78.52, *p* < 0.001, ΔCFI = 0.006) and Model 4 (Δχ^2^ (26) = 85.10, *p* < 0.001, ΔCFI = 0.008). In both cases, although the difference in χ^2^ from the configural model was statistically significant, the difference between the CFI values met the recommended cut-off criterion of 0.01 [52]. Using the CFI difference test as the criterion upon which to identify evidence of invariance, we concluded that the factor loadings operated similarly across groups in the three countries. These results confirm the role of self-identity in moderating the effects of intention antecedents on the intention–behavior relationship.

In both Model 3 and 4, subjective norm was more important in affecting the intention of the Low-SI group, implying that these people were prone to being more influenced by others (*p* < 0.001). On the other hand, the PBC effect was stronger in the Medium- (*p* < 0.001) and High-SI (*p* < 0.001) groups, suggesting that, for these groups, the perceived control over the adoption of a SuDiet in the near future had a stronger effect on intentions. Moreover, the Low-SI group exhibited a stronger prediction role for attitude on intention (*p* < 0.001). This indicates that individuals less interested in the sustainability of dietary behavior but with more favorable opinions of it are more likely to be willing to adopt a SuDiet in the future. Intention was the only significant predictor of self-perceived behavior in Model 3 across the three groups, exerting a stronger effect on the Medium-SI group. In Model 4, intention was able to explain behavior only in the High-SI group.

## 4. Discussion

The present observational study applied a theoretically driven survey built on the TPB to explain, for the first time, the intention to follow a SuDiet and its adoption in the near future, in a representative sample of adult residents in Italy. A set of SEM models were used to identify the most relevant drivers and barriers explaining behavioral intention and predicting behavior, which was assessed by both latent and observed variables. The former reflected the behavior adoption as perceived by the respondents, while the latter was the score of adherence to the MD. 

The findings show that intention better predicted the variance in behavior as self-perceived, compared to the derived measure (as calculated as the score of adherence to the MD), while PBC only significantly affected the observed measure. These results confirmed previous evidence showing that the TPB better explains self-reported behavioral measures compared to derived measures of behavior [31,54,55]. The discrepancy between self-perceived and more objective behavior might be explained by the non-complete overlapping between the two assessments. Secondly, respondents could have followed an alternative sustainable diet to the MD. This possibility is, however, mitigated by the higher MD scores found in subjects who agreed to having followed a SuDiet in the last three months compared to those who did not agree or agreed less with this statement [47]. Furthermore, the low compliance to the principle of compatibility [23,54] can be mentioned as an additional reason for the low level of explained variance obtained when the score of adherence to the MD acted as an endogenous variable. Indeed, it is worth noting that in this case the correspondence between the TPB constructs and the behavior of interest in terms of action, target, context, and time elements was not fully respected. Moreover, the self-perceived behavior assessment and TPB constructs were properly aligned as both used a seven-point scale of measurement, [54]. Conversely, the measurement of more objective behavior was based on a zero-to-nine score (for adherence to the MD) or on a five-point scale (for single food group consumption).

PBC plays a significant role in predicting the intention to follow a SuDiet. In other words, the higher the perceived ability to adopt a SuDiet, the higher the intention to actually adopt it. Attitude and subjective norm significantly contributed to form intention too, although to a lesser extent, indicating that one’s personal positive evaluation of the behavior and that of significant others had a direct impact on respondents’ motivations. These results are aligned with other studies showing PBC to be the most important predictor of intention compared to attitude and subjective norm [56,57,58,59]. Other researchers, however, have found attitude to be the predominant predictor of intention [43], attributing a weaker prediction role to PBC and subjective norm [60,61,62,63]. More specifically, the latter generally exerts a stronger role as a predictor of intention in adolescents [23,54,55]. 

As expected, when the consumption of single food groups was predicted, negative statistical regression coefficients were observed for intention to adopt a SuDiet in affecting meat product and sweet beverage consumption frequency. However, since a diet with a limited meat consumption was only conceptually associated with a SuDiet by 16% of the participants, this negative association does not seem to be consciously perceived by the respondents, or might even imply a deviation from rationality [24]. Intriguingly, among the top three reasons for limiting meat consumption, the environmental impact was less frequently indicated than health (25% vs. 60%) by flexitarian consumers [64], who seemed to underestimate the role of meat production, in particular beef, in contributing to environmental externalities. 

According to our findings, the moderating role of self-identity on the effects of intention antecedents highlighted the stronger relative importance of social norms in the respondents less interested in food sustainability (Low-SI group). On the contrary, the role played by third parties was less relevant in people who declared a higher interest in sustainability and were more likely to follow a sustainable diet. In addition, people in the high-SI group felt they had more control over their behavior, as supported by the strong correlation between these variables. The intention to adopt a SuDiet in this group was also strongly affected by changes in PBC. Concerning actual behavior performance, we found that a higher intention led to more pronounced MD adherence in the high-SI group. In other words, people in this group were more likely to be acting according to their motivations. This suggests that, for people more interested in diet sustainability, the facilitation of behavior and removal of barriers strengthens their intentions and makes the subsequent adoption of the Mediterranean dietary pattern more likely. In China, Wang et al. [43] found that the reasons cited for and against green consumption differently affect, respectively, attitudes and intentions; these paths might help to explain our findings, which showed different antecedents of behavior in the low-, medium- and high-SI groups. 

Based on the TPB postulates, to foster the behavior of interest, intervention strategies should act on the beliefs more strongly correlated with the constructs that have a higher positive impact on the behavior. Accordingly, interventions should strengthen the subject’s perceived control over their behavior through more informative labels on products, price reductions, and a wider variety of food in collective catering. To address these points, a reshaping of the food environment is highly recommended. For example, indications about the geographical origin and/or the environmental impact along the supply chain can be listed as options (see, e.g., [65]). These data are supported by a recently published pan-European survey [66], showing that the lack of clear labelling is one of the main barriers to sustainable eating: 57% of European consumers agree that sustainability information should be compulsory on food labels. Moreover, food services should offer a higher number of plant-based dishes and offer advice on how to compose nutritionally balanced menus [39,67]. 

Convenience, familiarity, and price have been indeed identified as motivational barriers able to prevent the adoption of a sustainable diet in French adults [67]. Consistently, price reduction due to subsidies can effectively increase the consumption of healthy food, such as fruit and vegetables [68]. 

The effect of subjective norms on behavioral intentions was stronger for the low-SI group; therefore, targeting the components linked to social influence on individual’s behavior should be a more effective strategy for this segment. In this context, the opinion of professional figures, such as doctors, nutritionists, or experts, as well as institutional campaigns addressed to the general population, could enhance individuals’ awareness of healthy and sustainable eating, mainly among those currently less interested in sustainability, with, in turn, a positive effect on the probability of people adopting a more eco-friendly diet. A comparable pattern has been shown in a German study [69], which found that being aware of climate change and humanity’s responsibility for it increased the probability of individuals adopting a low-carbon-emissive diet in younger generations, also including the consumption of in vitro meat products beside vegetarian or vegan alternatives. On a global level, alongside environmental awareness and price, higher education has been identified as a driver of plant-based dietary transition, conversely to income (in the short term), level of development, and globalization, which have been instead regarded as barriers [70]. In the present study, if adequately communicated to consumers, the support given to the local economy and to small and medium farmers and the food’s sensory attributes can be listed as the most relevant behavioral beliefs exerting an impact on behavior. The agreement of European consumers in spending higher prices for food products if a fair price is guaranteed to farmers has been also reported by the previously mentioned pan-European survey [66]. 

To the best of our knowledge, this research study pioneered the application of the TPB to examine the relative importance of behavioral precursors in explaining the intention to follow and the actual adoption of a sustainable diet by adults in Italy. Nevertheless, some limitations pertaining to the study design should be mentioned. First, although representative of the Italian population for some variables (gender, age, geographical area of residence), the sample was biased with respect to other variables (e.g., size of residence, education, household numbers), probably due to problems regarding recruitment and the self-selection of the participants. Secondly, the self-administered food frequency questionnaire may not have accurately collected information on actual food intake. Thirdly, the cross-sectional assessment of the behavior precluded the possibility of prospectively measuring eventual modifications in behavior over time and checking for potential changes in the role of behavioral predictors and the behavior itself. Finally, the cross-sectional measurement of behavior limited the compatibility principle based on time elements between the TPB constructs, which referred to the adoption of a SuDiet in the near future, and the behavior itself.

## 5. Conclusions

The present research explained and predicted the adoption of a sustainable diet and the factors affecting this behavior in a representative sample of adults in Italy, by applying the TPB. The behavior of interest was measured through two outcome variables: as a subjective measure and as a degree of adherence to the Mediterranean diet, considering the latter as a proxy for sustainable food consumption. Applying self-identity as a moderator of the effects of intention antecedents and the intention–behavior relationship suggested promising interventions for specific population groups. The obtained results support the need to address efforts in levering distinct motivations to drive dietary transition and develop intervention strategies tailored to adults as the target population. Beside the implementation of price mechanism strategies, educational initiatives mainly directed at increasing awareness about food and diet sustainability issues and addressed at strengthening the perceived control over food consumption at the individual level are recommended.

## Figures and Tables

**Table 1:** Percentage of respondents who associated the following concepts with the meaning of a sustainable diet. Participants could choose a maximum of three alternative answers.

	Respondents (%)
Diet with a low environmental impact that is respectful of biodiversity	58.8
Healthy and balanced diet	46.3
Diet including products whose purchase sustains and protects the workers in the agricultural sector	30.3
Diet based on seasonal foods	29.2
Diet based on local/or traditional products	24.7
Mediterranean diet	23.8
Diet with a limited meat consumption	15.5
Diet based on products with certifications linked to the area and/or method of production	14.8
Economic diet	5.7
Vegetarian diet	2.7
Vegan diet	2.5

**Table 2:** Median values (IR) of single items, assessed on a seven-point Likert scale, used to inform the TPB constructs displayed with indices of internal validity, reliability, and consistency of the same constructs.

	Median (IR)	λ	CR	AVE	α
Attitude (Adopting a SuDiet is…)	6.0 (5.3–6.8)		0.922	0.748	0.923
Useless/useful	6.0 (5.0–7.0)	0.861			
Unsatisfactory/satisfactory	6.0 (5.0–7.0)	0.921			
Unhealthy/healthy	6.0 (6.0–7.0)	0.801			
Unpleasant/pleasant	6.0 (5.0–7.0)	0.872			
Subjective norm (Most people…)	4.3 (3.5–5.0)		0.872	0.630	0.869
Important to me think I should adopt a SuDiet	4.0 (3.0–5.0)	0.771			
I esteem would approve of me adopting a SuDiet	5.0 (4.0–6.0)	0.779			
I respect will adopt a SuDiet	4.0 (4.0–5.0)	0.862			
Like me have adopted a SuDiet	4.0 (3.0–5.0)	0.758			
Perceived behavioral control	5.3 (4.5–6.0)		0.841	0.641	0.870
I believe I am able to adopt a SuDiet	5.0 (4.0–6.0)	0.938			
If I wanted to, I could adopt a SuDiet	5.0 (5.0–6.0)	0.763			
Whether I adopt a SuDiet is under my control	5.0 (5.0–6.0)	0.680			
Self-identity			-	-	-
I think of myself as a person interested in diet sustainability	5.0 (4.0–6.0)	-			
Intention	5.0 (4.3–6.0)		0.967	0.879	0.968
I intend to adopt a SuDiet in the near future	5.0 (4.0–6.0)	0.933			
I want to adopt a SuDiet in the near future	5.0 (4.0–6.0)	0.929			
I will adopt a SuDiet in the near future	5.0 (4.0–6.0)	0.945			
I plan to adopt a SuDiet in the near future	5.0 (4.0–6.0)	0.943			
Self-perceived behavior (I can say I have…)	4.7 (3.7–5.3)		0.836	0.632	0.833
Adopted a SuDiet within the last 3 months	4.0 (3.0–5.0)	0.876			
Limited my consumption of animal-based products in the last 3 months	5.0 (3.0–5.8)	0.811			
Mostly consumed local and/or seasonal foods in the last 3 months	5.0 (4.0–6.0)	0.687			

Note: internal validity, reliability, and consistency of the TPB constructs are respectively expressed as factor loadings (λ), composite reliability (CR), average variance extracted (AVE), and Cronbach’s α.

**Table 3:** Spearman’s rank-order correlation (ρ) between TPB constructs and convergent validity of the TPB constructs, expressed as the square root of the average variance extracted (AVE) of each construct (reported in bold).

	Attitude	SN	PBC	Intention	SP-Behavior	Behavior *
Attitude	**0.86**	0.49	0.61	0.68	0.52	0.32
SN		**0.79**	0.51	0.59	0.49	0.27
PBC			**0.80**	0.77	0.54	0.35
Intention				**0.97**	0.67	0.36
SP-Behavior					**0.79**	0.39
Behavior *						1.00

Note: * Adherence to the MD; all correlations are significant at 99.9% level (*p* < 0.001). PBC: perceived behavioral control; SP: self-perceived; PBC: perceived behavioral control; SN: subjective norm.

**Table 4:** Correlations between salient beliefs and attitude, subjective norm (SN), perceived behavioral control (PBC), intention, self-perceived (SP) behavior, and actual behavior (adherence to the MD).

**Beliefs Categories**	**TPB Constructs**			
**Behavioral Beliefs**	**ATT**	**INT**	**SP-Behav.**	**Behav.**
Positive impact on health	0.477	0.469	0.384	0.261
Positive effect on environment	0.500	0.485	0.368	0.250
Adoption of an ethical behavior	0.514	0.482	0.366	0.244
Satisfaction from a sensory perspective	0.555	0.500	0.391	0.244
Food habit modification	0.388	0.329	0.319	0.182
Improvement of culinary skills	0.407	0.364	0.329	0.191
Support the local economy	0.498	0.444	0.424	0.245
Support small-/medium-sized farmers	0.507	0.481	0.421	0.262
**Normative (injunctive) beliefs**	**SN**	**INT**	**SP-Behav.**	**Behav.**
Partner	0.582	0.472	0.350	0.210
Family	0.608	0.465	0.373	0.229
Dear friends	0.597	0.384	0.330	0.168
Doctors/nutritionists/experts	0.541	0.426	0.304	0.157
Institutions	0.524	0.364	0.294	0.160
**Normative (descriptive) beliefs**	**SN**	**INT**	**SP-Behav.**	**Behav.**
Partner	0.604	0.541	0.389	0.257
Family	0.657	0.558	0.461	0.283
Dear friends	0.634	0.463	0.403	0.249
**Control beliefs**	**PBC**	**INT**	**SP-Behav.**	**Behav.**
Informative labels on the products	0.482	0.501	0.425	0.294
Price reduction	0.359	0.376	0.363	0.201
Free time available	0.321	0.367	0.373	0.217
Not being alone	0.345	0.389	0.360	0.190
Food variety in collective catering	0.418	0.449	0.365	0.203

Note: Spearman’s rank-order correlation (ρ). All correlations are significant at *p* < 0.001. ATT: attitude; INT: intention; PBC: perceived behavioral control; SN: subjective norm.

**Table 5:** SEM relating attitude, subjective norms (SN), and perceived behavioral control (PBC) to intention, self-perceived (SP) behavior, and actual behavior (adherence to the MD).

**Model 1**	**B**	**SE**	**ß**	**R^2^**
Attitude → Intention	0.252 ***	0.037	0.210	0.779
SN → Intention	0.233 ***	0.037	0.214	
PBC → Intention	0.592 ***	0.047	0.568	
Intention → SP-Behavior	0.761 ***	0.070	0.669	0.543
PBC → SP-Behavior	0.094	0.073	0.079	
**Model 2**	**B**	**SE**	**ß**	**R^2^**
Attitude → Intention	0.247 ***	0.037	0.206	0.778
SN → Intention	0.227 ***	0.037	0.207	
PBC → Intention	0.603 ***	0.048	0.576	
Intention → Behavior	0.262 **	0.097	0.193	0.130
PBC → Behavior	0.258 *	0.104	0.182	

Note: *** *p* < 0.001, ** *p* < 0.01, * *p* < 0.05. Model 1 fit indices: χ^2^ (DF) = 399.16(121) ***, TLI = 0.974; CFI = 0.979; RMSEA (90% C.I.) = 0.052 (0.047–0.058); SRMR = 0.033. Model 2 fit indices: χ^2^ (DF) = 240.63(91) ***, TLI = 0.983; CFI = 0.987; RMSEA (90% C.I.) = 0.044 (0.038–0.051); SRMR = 0.026. Unstandardized coefficients (B) and standardized coefficients (ß) refer to the paths from exogenous to the endogenous variables (i.e., from attitude, SN, and PBC to intention and from PBC to (SP)-behavior), as well as to the path between endogenous variables (i.e., from intention to (SP)-behavior). SE: standard error.

**Table 6:** TPB construct values referring to self-identity (SI) groups and total sample.

	Total (*n* = 838)	Low SI (*n* = 245)	Medium SI (*n* = 300)	High SI (*n* = 293)	*p* Value
Attitude	6.0 (5.3–6.8)	5.0 (4.0–5.8)	6.0 (5.5–5.6)	6.5 (5.8–7.0)	<0.001
Subjective norm	4.3 (3.5–5.0)	3.5 (2.5–4.0)	4.5 (4.0–4.8)	4.5 (4.0–5.3)	<0.001
PBC	5.3 (4.5–6.0)	4.3 (3.8–5.0)	5.3 (4.8–4.8)	5.8 (5.0–6.3)	<0.001
Intention	5.0 (4.3–6.0)	4.0 (3.0–4.3)	5.0 (4.8–5.5)	5.8 (5.0–6.5)	<0.001
Behavior	4.0 (3.0–5.0)	3.0 (2.0–4.0)	4.0 (3.0–5.0)	4.0 (3.0–6.0)	<0.001
SP-Behavior	4.7 (3.7–5.3)	3.3 (2.3–4.0)	4.7 (3.7–5.0)	5.0 (4.3–5.7)	<0.001

Note: data are expressed as median (IR). Comparison between Low-, Medium-, and High-SI groups and TPB constructs; non-parametric Kruskal Wallis test used for independent samples. PBC: perceived behavioral control; SP: self-perceived.

**Table 7:** SEM multi-group analysis showing unstandardized regression coefficients (B) and standard error (SE).

**Model 3**	**Low SI**		**Medium SI**		**High SI**	
	**B**	**SE**	**B**	**SE**	**B**	**SE**
Attitude → Intention	0.192 ***	0.055	0.100	0.054	0.160 *	0.079
SN → Intention	0.558 ***	0.091	0.252 ***	0.063	0.056	0.032
PBC → Intention	0.143 **	0.053	0.439 ***	0.092	0.580 ***	0.088
Intention → SP-Behavior	0.310 ***	0.065	0.478 ***	0.108	0.421 **	0.144
PBC → SP-Behavior	−0.006	0.028	0.002	0.084	0.148	0.127
**Model 4**	**Low SI**		**Medium SI**		**High SI**	
	**B**	**SE**	**B**	**SE**	**B**	**SE**
Attitude → Intention	0.190 ***	0.055	0.093	0.055	0.169 *	0.078
SN → Intention	0.553 ***	0.091	0.236 ***	0.063	0.061	0.031
PBC → Intention	0.149 **	0.054	0.470 ***	0.095	0.564 ***	0.087
Intention → Behavior	0.004	0.099	0.008	0.244	0.700 *	0.292
PBC → Behavior	0.078	0.059	0.359	0.238	−0.074	0.257

Note: *** *p* < 0.001, ** *p* < 0.01, * *p* < 0.05. Model 3 fit indices: χ^2^ (DF) = 698.52(363) ***, TLI = 0.947; CFI = 0.958; RMSEA (90% C.I.) = 0.033 (0.030–0.037); SRMR = 0.057. Model 4 fit indices: χ^2^ (DF) = 444.77(273) ***, TLI = 0.968; CFI = 0.976; RMSEA (90% C.I.) = 0.027 (0.023–0.032); SRMR = 0.047. Unstandardized coefficients (B) and standardized coefficients (ß) refer to the paths from exogenous to the endogenous variables (i.e., from attitude, SN and PBC to intention and from PBC to (SP)-behavior), as well as to the path between endogenous variables (i.e., from intention to (SP)-behavior). PBC: perceived behavioral control; SE: standard error; SN: subjective norm; SP: self-perceived.

## Data Availability

The data presented in this study are available on request from the corresponding author.

## Supplementary Materials

The following supporting information can be downloaded at: https://www.mdpi.com/article/10.3390/nu15122784/s1. Table S1: Detailed description of the TPB core constructs measured in the study; Table S2: Anthropometric variables, socio-demographic characteristics, and food-related habits of respondents (*n* = 838) and national absolute data (expressed as thousands); Table S3: Median values (IR) of behavioral, normative, and control beliefs; Table S4: Unstandardized coefficients (B) and standard error (SE) of intention and PBC as predictors of the consumption frequency of single food groups, and the explained variance (R^2^).
