# Supplementary material for: Predicting the Adoption of a Sustainable Diet in Adults: A Cross-Sectional Study in Italy
Source: Nutrients. 2023 Jun 17;15(12):2784. doi: 10.3390/nu15122784 (PMC10304942; doi:10.3390/nu15122784)
Supplement: Supplementary file 1 [file nutrients-15-02784-s001.zip › nutrients-2421218-SI.pdf]

Supplemental

Table S1. Detailed description of the TPB core constructs measured in the study.

TPB constructs	Description
**Attitude**	It was directly measured by using four polar adjectives (e.g., not useful at all-very useful). Indirect attitude measures were calculated by multiplying eight outcome expectancy items representing the perceived likelihood that salient outcomes will occur if a SuDiet will be adopted by the subject (e.g., if I adopt a SuDiet in the next future, it will contribute to reduce the environmental impact: very unlikely/very likely), with the corresponding outcome evaluation items measured on unipolar scale anchored by desirability (e.g., reducing environmental impact is for me not desirable at all/very desirable).
**Subjective norm**	Injunctive and descriptive normative beliefs were considered. The formers were used to obtain an indirect measure of subjective norm by multiplying five injunctive beliefs referent items (e.g., my family think I should adopt a SuDiet in the next future: very unlikely/very likely), with the corresponding motivations to comply items (e.g., I'm interested if my family approve or disapprove my choices when it comes to food: not at all/very much). Three descriptive normative beliefs were instead used to assess the probability that relevant others (i.e., my family/partner/dear friends) will enact the behaviour in the next future. In addition, subjective norm was directly measured by four items (e.g., most of the people I value would approve I adopt a sustainable diet).
**Perceived behavioural control**	Direct measures consisted of four items (e.g., I think that the adoption of a SuDiet in the next future is under my control: absolutely no/absolutely yes), among which one was used to measure self-efficacy (e.g., I believe that I am able to adopt a SuDiet in the next future: absolutely not/absolutely yes). In parallel, the indirect measure was obtained by multiplying five control belief factors (e.g., I will have more free time in the next future: very unlikely/very likely), with the corresponding control belief power items (e.g., having more free time would allow me to adopt a sustainable diet: very unlikely/very likely). Control beliefs assessed participants' perceptions about their ability to adopt a SuDiet in the next future despite intrinsic and extrinsic barriers.
**Behavioural intention**	It was measured with four items (e.g., I intend to adopt a SuDiet in the next future: absolutely no/absolutely yes).

Table S2. Anthropometric variables, socio-demographic characteristics, and food-related habits of respondents (n=838) and national absolute data (expressed as thousands).

	Sample N (%)	Italian population N (%)
Gender		
Female	434 (51.8)	19,405.3 (50.1)
Male	404 (48.2)	19,354.2 (49.9)
Age (years)		
18–24	89 (10.6)	4,158.4 (11.9)
25–34	157 (18.7)	6,643.6 (19.0)
35–44	198 (23.6)	8,373.1 (24.0)
45–54	209 (24.9)	7,849.3 (22.5)
55–65	185 (22.1)	7,911.9 (22.7)
BMI (kg/m²)		
<25.0	533 (63.6)	(56.2)
≥25.0	305 (36.4)	(43.8)
Nationality		
Italian	818 (97.6)	5,542.1 (91.7)
Other	20 (2.4)	5,047.0 (8.3)
Geographical area of residence		
Nord-West	220 (26.3)	10,185.0 (26.1)
Nord-East	168 (20.1)	7,396.1 (19.0)
Centre	167 (19.9)	7,706.7 (19.8)
South	192 (22.9)	9,305.4 (23.9)
Islands	91 (10.9)	4,420.6 (11.3)
Size of residence (number of inhabitants)		
< 50,000	148 (17.7)	41.546,2 (68.9)*
≥ 50,000	690 (82.3)	18.813,3 (31.1) ¥
Education		
Primary/secondary	510 (60.9)	44.936,9 (80,1)
Tertiary	328 (39.1)	6,271.0 (11.2)
Occupation		
Employee	574 (68.5)	22,850.8 (59.4)
Unemployed/retired/student	264 (31.5)	5,636.4 (6.6)⊕
Monthly household net income (€)◊		
< EUR 900	49 (5.9)	n.a.
EUR 900 – 1,499	144 (17.2)	n.a.
EUR 1,500 – 2,499	267 (31.9)	n.a.
EUR 2,500 – 3,499	176 (21.0)	n.a.
≥3,500	118 (14.1)	n.a.
Do not wish to tell/do not know	84 (10.0)	n.a.
Household members (n)		
1-2	258 (30.8)	15.529 (60.4)
≥3	580 (69.2)	10.186 (39.6)
Responsibility in food purchase		
Main responsible	601 (71.7)	(48.1)
No main responsible	237 (28.3)	(51.9)

Responsibility in meal preparation		
Main responsible	529 (63.1)	(32.7)
No main responsible	309 (36.9)	(67.3)[#]
Frequency of eating out		
≤ 1 time/week	491 (58.6)	(54.5)[♦]
2-4 times/week	249 (29.7)	(19.4)[♠]
≥ 5 times/week	98 (11.7)	(26.1)[•]

Note: Data referred to Italian population has been retrieved from Eurostat (gender, 2018; age, 2018; geographical area of residence, 2018; BMI, 2014; occupation, 2019: [⊕] data refer only to the unemployed population), Istat (residence size, 2019: [*]<59,999 inhabitants, [¥] ≥60,000 inhabitants; education, 2011; nationality, 2016; household members, 2019), Italian Federation of Public Enterprises, FIPE (responsibility of food purchase, 2018; responsibility of food preparation, 2018: Italian adults who stated to cook every day, [#]Italian adults who stated to cook sometimes/never cook; frequency of eating out, 2018: [♦]Italian adults who stated to eat out ≤ 1 or 2-3 meals per month, [♠]Italian adults who stated to eat out 2/3 meals per week, [•]Italian adults who stated to eat out 4/5 meals per week); [◊]Median equivalised net income for Italy (18-64 years) in 2019 corresponded to EUR 17,165, i.e., EUR 1.430 per month (Source: Eurostat); n.a.: not available.

Table S3. Median values (IR) of behavioural, normative and control beliefs.

**Beliefs categories**	**Beliefs components**		
Behavioural	(1) #	(2) #	(1)*(2)
Positive impact on health	2.0 (1.0-2.0)	2.0 (1.0-3.0)	3.0 (1.0-6.0)
Positive effect on environment	2.0 (1.0-2.0)	2.0 (1.0-2.0)	3.0 (1.0-4.0)
Adoption of an ethical behaviour	2.0 (1.0-2.0)	2.0 (1.0-2.0)	2.0 (1.0-4.0)
Satisfaction from a sensory perspective	1.0 (0.0-2.0)	2.0 (1.0-2.0)	2.0 (0.0-4.0)
Food habits modification	1.0 (0.0-2.0)	1.0 (0.0-2.0)	1.0 (0.0-2.0)
Improvement of culinary skills	1.0 (0.0-2.0)	1.0 (1.0-2.0)	1.0 (0.0-4.0)
Support to local economy	2.0 (1.0-2.0)	1.0 (1.0-2.0)	2.0 (1.0-4.0)
Support to small/medium size farmers	2.0 (1.0-2.0)	1.0 (1.0-2.0)	2.0 (1.0-4.0)
Normative (injunctive)	(3) #	(4)	(3)*(4)
Partner	0.0 (0.0-1.0)	5.0 (4.0-6.0)	0.0 (0.0-6.0)
Family	0.0 (-1.0-1.0)	5.0 (4.0-6.0)	0.0 (-2.0-6.0)
Dear friends	0.0 (-1.0-1.0)	4.0 (3.0-5.0)	0.0 (-3.0-4.0)
Doctors/nutritionists/experts	1.0 (0.0-2.0)	5.0 (4.0-6.0)	2.0 (0.0-8.0)
Institutions	0.0 (-1.0-1.0)	4.0 (3.0-5.0)	0.0 (-2.0-5.0)
Normative (descriptive)	(5)	(4)	(5)*(4)
Partner	1.0 (0.0-2.0)	5.0 (4.0-6.0)	3.0 (0.0-7.0)
Family	1.0 (0.0-2.0)	5.0 (4.0-6.0)	1.0 (0.0-6.0)
Dear friends	0.0 (-1.0-1.0)	4.0 (3.0-5.0)	0.0 (-2.0-5.0)
Control	(6)	(7)	(6)*(7)
Informative labels on the products	5.0 (5.0-6.0)	2.0 (1.0-2.0)	8.0 (5.0-12.0)
Price reduction	5.0 (4.0-6.0)	2.0 (1.0-2.0)	6.0 (4.0-12.0)
Free time available	5.0 (4.0-5.0)	1.0 (0.0-2.0)	5.0 (0.0-10.0)
Not being alone	5.0 (4.0-6.0)	1.0 (0.0-2.0)	5.0 (0.0-10.0)
Food variety in collective catering	5.0 (4.0-6.0)	1.5 (1.0-2.0)	6.0 (4.0-12.0)

Note: #converted to bipolar scale (-3; +3); (1): Behavioural belief strength; (2): outcome evaluation; (3): injunctive belief strength; (4): motivation to comply; (5): descriptive belief strength; (6): control strength; (7): power control factors.

Table S4. Unstandardized coefficients (B) and standard error (SE) of intention and PBC as predictors of the consumption frequency of single food groups, and explained variance (R^2).

	Intention		PBC		R^2
	B	SE	B	SE	
Wholegrain pasta or rice	-0.019	0.051	0.073	0.054	0.007
Wholegrain bread and substitutes	0.007	0.052	0.099	0.057	0.021
Pulses	0.117	0.060	0.125*	0.064	0.076
Milk and yoghurt	0.049	0.059	0.095	0.063	0.030
White meat	-0.118**	0.045	0.065	0.048	0.015
Red meat, meat products	-0.217***	0.048	0.070	0.052	0.062
Fish or sea foods	-0.058	0.064	0.224***	0.070	0.043
Vegetables, all types	0.146**	0.055	0.086	0.058	0.079
Fruit, all types fresh and fresh juices	0.178**	0.062	0.080	0.066	0.083
Nuts	0.155	0.081	0.137	0.087	0.067
Olive oil to cook and to dress	0.009	0.044	0.100*	0.048	0.031
Butter, margarine or cooking cream	-0.039	0.044	0.030	0.047	0.001
Carbonated and/or sugar-sweetened beverage	-0.128*	0.060	-0.003	0.062	0.026
Sweets	-0.034	0.064	-0.038	0.068	0.006
Wine	0.020	0.051	-0.031	0.055	0.001

Note: The positive or negative B value indicated the extend by which each single food consumption respectively increased or decreased when intention or PBC went up by 1 unit. $^*p < 0.05$; $^{**}p < 0.01$; $^{***}p < 0.001$. PBC: perceived behavioural control.
